# An Algorithm to Optimize the Micro-Geometrical Dimensions of Scaffolds with Spherical Pores

**DOI:** 10.3390/ma13184062

**Published:** 2020-09-13

**Authors:** Óscar Libardo Rodríguez-Montaño, Carlos Julio Cortés-Rodríguez, Antonio Emmanuele Uva, Michele Fiorentino, Michele Gattullo, Vito Modesto Manghisi, Antonio Boccaccio

**Affiliations:** 1 Departamento de Ingeniería Mecánica y Mecatrónica, Universidad Nacional de Colombia, 111321 Bogotá, Colombia; olrodriguezm@unal.edu.co (Ó.L.R.-M.); cjcortesr@unal.edu.co (C.J.C.-R.); 2 Dipartimento di Meccanica, Matematica e Management, Politecnico di Bari, 70125 Bari, Italy; antonio.uva@poliba.it (A.E.U.); michele.fiorentino@poliba.it (M.F.); michele.gattullo@poliba.it (M.G.); vitomodesto.manghisi@poliba.it (V.M.M.)

**Keywords:** geometry optimization, computational mechanobiology, bone tissue engineering, python code, parametric CAD (Computer Aided Design) model

## Abstract

Despite the wide use of scaffolds with spherical pores in the clinical context, no studies are reported in the literature that optimize the micro-architecture dimensions of such scaffolds to maximize the amounts of neo-formed bone. In this study, a mechanobiology-based optimization algorithm was implemented to determine the optimal geometry of scaffolds with spherical pores subjected to both compression and shear loading. We found that these scaffolds are particularly suited to bear shear loads; the amounts of bone predicted to form for this load type are, in fact, larger than those predicted in other scaffold geometries. Knowing the anthropometric characteristics of the patient, one can hypothesize the possible value of load acting on the scaffold that will be implanted and, through the proposed algorithm, determine the optimal dimensions of the scaffold that favor the formation of the largest amounts of bone. The proposed algorithm can guide and support the surgeon in the choice of a “personalized” scaffold that better suits the anthropometric characteristics of the patient, thus allowing to achieve a successful follow-up in the shortest possible time.

## 1. Introduction

One of the main issues recently investigated in the field of bone tissue engineering and that has received substantial attention is the identification of the optimal geometry of bony tissue scaffolds to support the numerous cellular activities involved in bone formation and regeneration [1]. Scaffolds are porous structures that mainly perform a dual function: transporting nutrients, waste, and oxygen, and a structural function consisting of transferring the load to the cells and regenerated tissues occupying their pores and to the adjacent tissues where they are implanted [2,3]. A large number of porous topologies have been studied from both the theoretical and the experimental point of view, but there is not yet a consensus between researchers regarding the geometry that the “optimal” scaffold should possess to maximize the amounts of regenerated bone [4]. However, some “general” guidelines are commonly accepted in the literature such as the range of the dimensions that pores have to possess to favor the regeneration process [5].

In general, bone tissue scaffolds can be classified into two principal categories: irregular and regular. Regular scaffolds are fabricated using advanced manufacturing processes such as additive layer manufacturing (ALM) that allow controlling with high precision the specific dimension of the single unit cell the scaffold is made from. The irregular scaffolds are fabricated with conventional physical-chemical processes that allow controlling the average dimensions of the scaffold microarchitecture only on a statistical base [6]. A typical advantage of regular structures is the regularity of the scaffold domain that implies the regularity of the physical environment and hence the regularity of the mechanical stimulus acting on the regenerating tissue.

A very interesting scaffold topology is that including spherical pores. It is commonly known that the adhesion and differentiation of stem cells take place more easily on curved surfaces, especially on concave surfaces [5,7]. Scaffold topologies including spherical pores were recently produced with ALM techniques [8]. Spherical pores are also included in previously explored scaffold geometries such as FCC (face-centered cubic), BCC (body-centered cubic) [9,10], and Schwartz-P primitives [11,12,13]. However, no studies are reported in the literature optimizing the geometry of scaffolds with spherical pores, with the scope of maximizing the amounts of neo-formed bone. Here we aim to bridge this gap. We modeled the scaffold and the tissues occupying it as biphasic poroelastic materials and computed the biophysical stimulus acting on the tissue inside the scaffold pores according to the model of Prendergast et al. [14], as a function of the octahedral shear strain and the interstitial fluid flow. The objective of this study was to identify the optimal geometrical parameters of a regular scaffold with spherical pores and cylindrical interconnections that maximize the amounts of neo-formed bone. We found that this scaffold topology is particularly suited to bear shear loads. The proposed model fits well the requirements of so-called Precision Medicine (i.e., the branch of Medicine that studies personalized medical solutions for the specific requirements of the patient) and tries to answer the question about the optimal scaffold micro-geometry to achieve a successful follow-up in the shortest possible time.

## 2. Materials and Methods

### 2.1. Unit Cell Geometry

**Figure 1:**
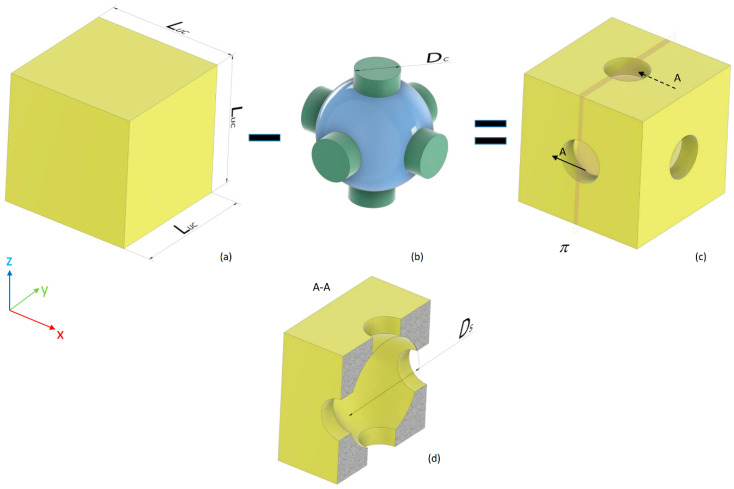
To build the scaffold unit cell (**c**), a boolean subtraction was carried out between a cubic volume (side *L_uc_* = *L*/4) (**a**) and the volume of a sphere (highlighted in blue) with cylinders (highlighted in green) oriented orthogonally according to the coordinate axes (**b**). The section A-A view (**d**) with the plane π (**c**), shows how the unit cell is interiorly made.

**Figure 2:**
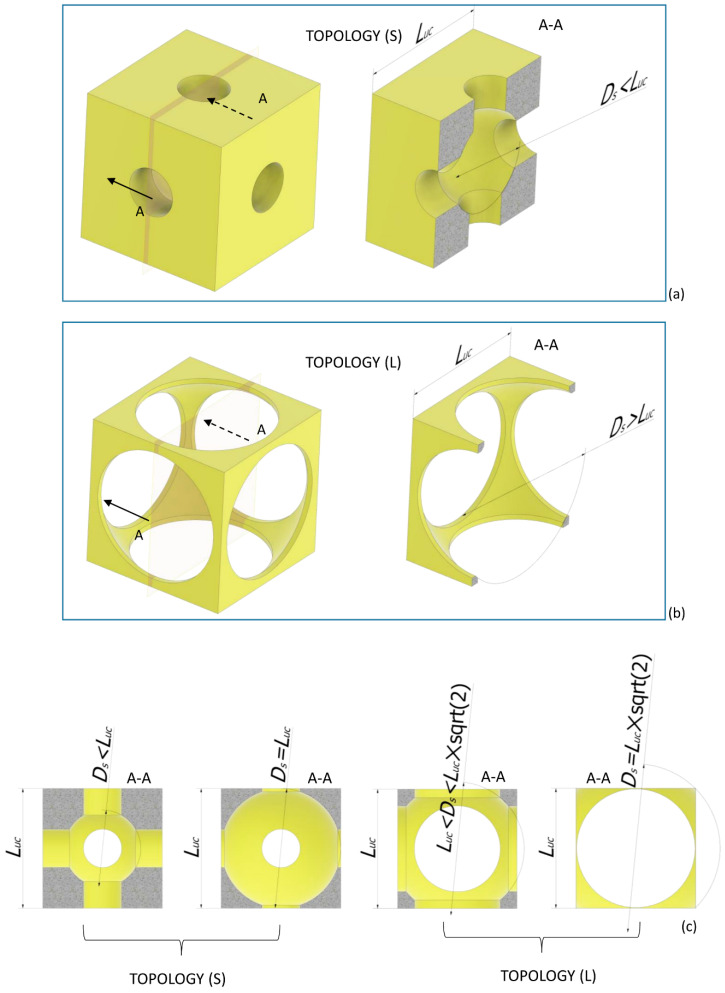
Two different topologies can be built for the scaffold unit cell: “small” (S) (**a**) and “large” (L) (**b**). Topology (S) includes a spherical surface with 0 < *D_s_* ≤ *L_uc_* (**c**); Topology (L) includes a spherical surface with *L_uc_* < *D_s_* ≤ $\sqrt{2}$ × *L_uc_* (**c**).

The parametric model of a scaffold occupying a cubic volume of side *L =* 2.548 mm and including 4 × 4 × 4 = 64 unit cells was developed. The same scaffold dimensions were utilized in previous studies [15,16]. The general purpose software Abaqus (version 6.12, Dassault Systèmes, Vélizy-Villacoublay, France) was utilized for both the parametric geometry modeling and the finite element analysis. Each unit cell is a hexahedron with a spherical cavity and cylindrical interconnections oriented along the orthogonal directions of the coordinate axes. It can be obtained as a Boolean subtraction of the volume of a sphere with cylinders from a cubic volume with the side *L_uc_* = *L*/4 (Figure 1). Depending on the diameter of the spherical surface *D_s_*, two different unit cell topologies can be designed: a “small” (S) topology where 0 < *D_s_* ≤ *L_uc_* and a “large” (L) topology where *L_uc_* < *D_s_* < *L_uc_* × $\sqrt{2}$ (Figure 2). Obviously, spherical diameters *D_s_* > *L_uc_* × $\sqrt{2}$ are not allowed, as the geometry deriving from such an assumption would lead to a scaffold unit cell completely different with respect to that hypothesized. Regarding the diameters of cylinders *D_c_*, other constraints must be respected depending on the specific topology. In the case of Topology (S), the diameter of cylinders must satisfy the following inequality:

(1) $$0\;<\;D_{c}\;\leq \;D_{s}/\sqrt{2},$$

**Figure 3:**
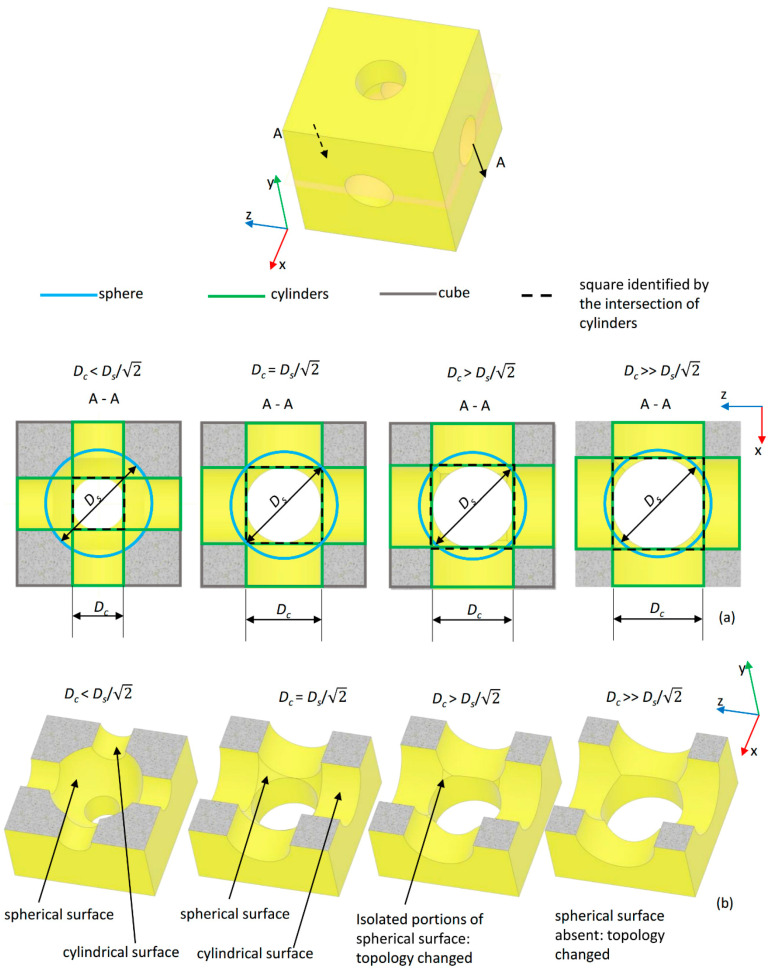
(**a**) Section views—in the plane x–z—of the scaffold unit cell (topology (S) with indicated edges of the primitives (cube, cylinders, and sphere) utilized. When the square obtained by the intersection of the cylinders touches with its vertices, the spherical edge (in blue), the limit condition *D_c_* = *D_s_*/$\sqrt{2}$ is reached. For *D_c_* > *D_s_*/$\sqrt{2}$, the topology of the unit cell changes. (**b**) Section views—in the three-dimensional space—of the unit cell obtained for different values of *D_s_* and *D_c_*.

In the section views obtained with a plane cutting the unit cell in half (Figure 3a), the figure of a square (represented with a dashed line, Figure 3) can be traced as the intersection of the edges of the cylinders. If this square is included within the edge of the spherical surface (highlighted in blue, Figure 3), the inequality (1) is verified. Inside the unit cell, a unique spherical surface can be identified that is interrupted by the cylindrical surfaces (Figure 3b). When the vertices of the square touch the spherical edge, the condition

(2) $$D_{c}\;=\;D_{s}/\sqrt{2},$$

is reached. Finally, when the vertices of the square go beyond the spherical edges, only isolated (i.e., *D_c_* > *D_s_*/$\sqrt{2}$) or no (i.e., *D_c_* >> *D_s_*/$\sqrt{2}$) portions of spherical surface can be identified, and the geometry of the unit cell changes completely with respect to that hypothesized, which leads to the change in the scaffold connectivity.

In the case of Topology (L), the diameter of cylindrical surfaces *D_c_* must satisfy the following inequality

(3) $$\sqrt{\left(D_{s}^{2}-L_{uc}^{2}\right)}<D_{c}\leq D_{s}/\sqrt{2}\;,$$

**Figure 4:**
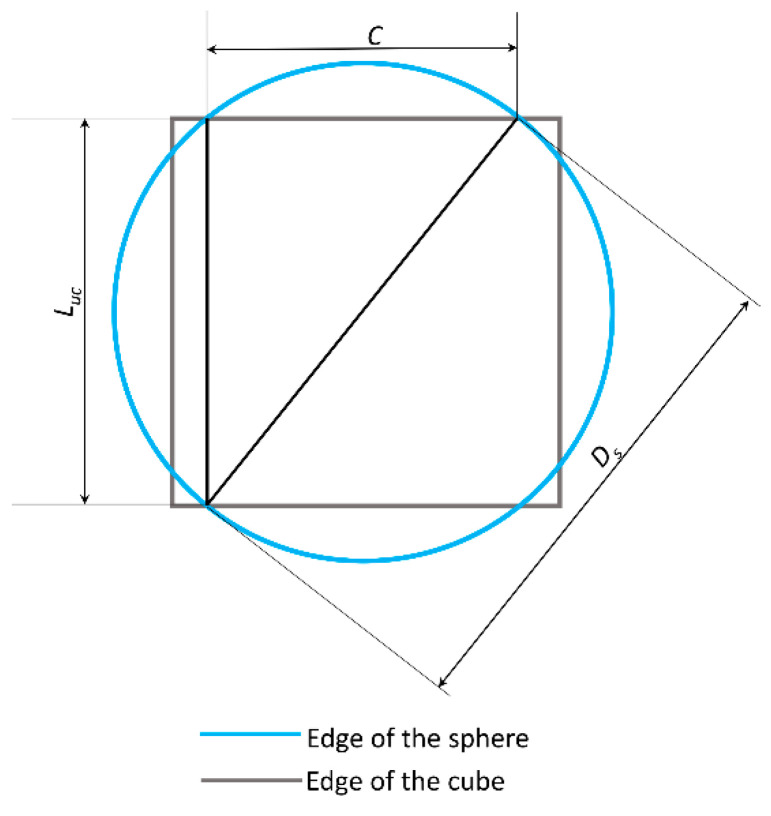
Schematic utilized to determine the equation constraint that the diameter of cylinders *D_c_* must satisfy in the case of Topology (L).

In fact, to guarantee the “coherence” of the hypothesized scaffold geometry, the cylindrical diameter must be greater than the length of the chord *C* obtained by the intersection of the spherical edge with the edge of the cylindrical surface (Figure 4). The length of the chord is given by

(4) $$C=\sqrt{\left(D_{s}^{2}-L_{uc}^{2}\right)}\;,$$

The considerations regarding the figure of the square that can be traced in the section view as the intersection of the cylindrical edges continue to remain valid also in the case of the Topology (L) and, consequently, lead to define the upper limit for *D_c_* that must be *D_c_* ≤ *D_s_*/$\sqrt{2}$. Table 1 summarizes the constraint equations that *D_s_* and *D_c_* must satisfy to guarantee that the unit cell geometry remains the same, thus conserving its “intrinsic” coherence, for the variable values that *D_s_* and *D_c_* can assume.

### 2.2. Scaffold Model and Applied Boundary and Loading Conditions

**Figure 5:**
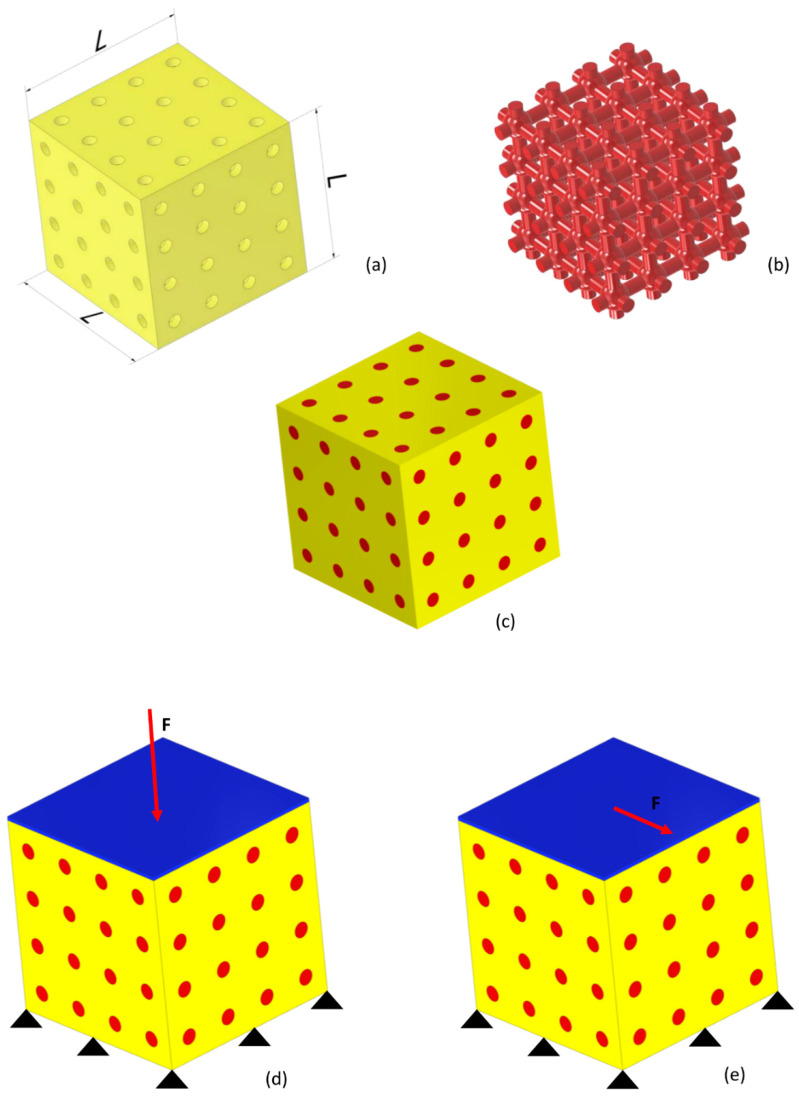
The CAD models of scaffold (**a**) and granulation tissue (**b**) were assembled to generate the model (**c**) utilized in the study. Two different boundary and loading conditions were hypothesized to act on the model: a compression load **F** (|**F**| = *F_UA_* × *L* × *L*) on the upper surface and an encastre on the lower one (**d**); a shear load **F** (|**F**| = *F_UA_* × *L* × *L*) on the upper surface and an encastre on the lower surface (**e**).

The unit cell described above was mirrored with respect to different planes and replicated 64 times to generate the geometry of the entire scaffold (Figure 5). The model includes also the granulation tissue, highlighted in red (Figure 5), occupying the scaffold pores. Both the scaffold and the granulation tissue were modeled as biphasic poroelastic materials with the same material properties (Table 1) as those utilized in previous studies [15,17,18]. 

A rigid plate (highlighted in blue, Figure 5d,e) was fixed at the upper face of the scaffold-granulation tissue system using a tie constraint to uniformly transfer the load. A tie constraint between the scaffold and granulation tissue was also established to prevent any relative displacement between these two materials. On the bottom surface of the model, an encastre boundary condition was fixed, while for the outer surfaces of the granulation tissue, a pore pressure equal to zero was set to allow, according to Byrne et al. [19], the free exudation of fluid. Two different loading conditions were hypothesized: a compression (Figure 5d) and a shear (Figure 5e) load. The values of load per unit area *F_UA_* hypothesized in this study were the same as those utilized in a previous article [16]: in the case of compression load, 0.05, 0.1, 0.5, 1.0, and 1.5 MPa, and in the case of shear load, 0.01, 0.05, 0.1, 0.2, and 0.5 MPa. C3D4P tetrahedral elements available in Abaqus^®^ were used to discretize the model. The average element size and the maximum deviation factor were set at 50 μm and 0.01, respectively.

A python script was generated that allows automatically (i) building the scaffold and the granulation tissue geometry; (ii) applying the boundary and the loading conditions; (iii) discretizing the model into finite elements; and (iv) running the finite element analyses. This script was then incorporated within an optimization code written in Matlab (Version R2016b, MathWorks, Natick, MA, USA) that, based on mechanobiological criteria deriving from the model of Prendergast et al. [14], allows the optimal scaffold geometry to be predicted.

### 2.3. A Brief Outline of the Mechano-Regulation Model Implemented to Determine the Scaffold Optimal Geometry

Once the scaffold is implanted in the region with bone deficiency, mesenchymal stem cells (MSCs) migrate from the adjacent tissues, thus invading the scaffold. Therefore, MSCs start their differentiation process. The model of Prendergast et al. [14] assumes that the biophysical stimulus *S* that triggers the differentiation process in the fracture domain is a function of the octahedral shear strain and of the interstitial fluid flow acting on the mesenchymal tissue. Depending on the values that *S* assumes, differentiation into different phenotypes, such as fibroblasts, chondrocytes, or osteoblasts, will be stimulated. The ranges of the biophysical stimulus *S* that determine the fate of the MSCs are described in the following inequalities:

*S > 3* → Fibroblasts (Fibrous tissue) 1 < *S* < 3 → Chondrocytes (Cartilage) 0.53 < *S* < 1 → Osteoblasts (Immature bone) 0.01 < *S* < 0.53 → Osteoblasts (Mature bone) 0 < *S* < 0.01 → Bone resorption (5)

Further details on the mechano-regulation algorithm can be found in previous studies [20,21].

### 2.4. Optimization Algorithm

**Figure 6:**
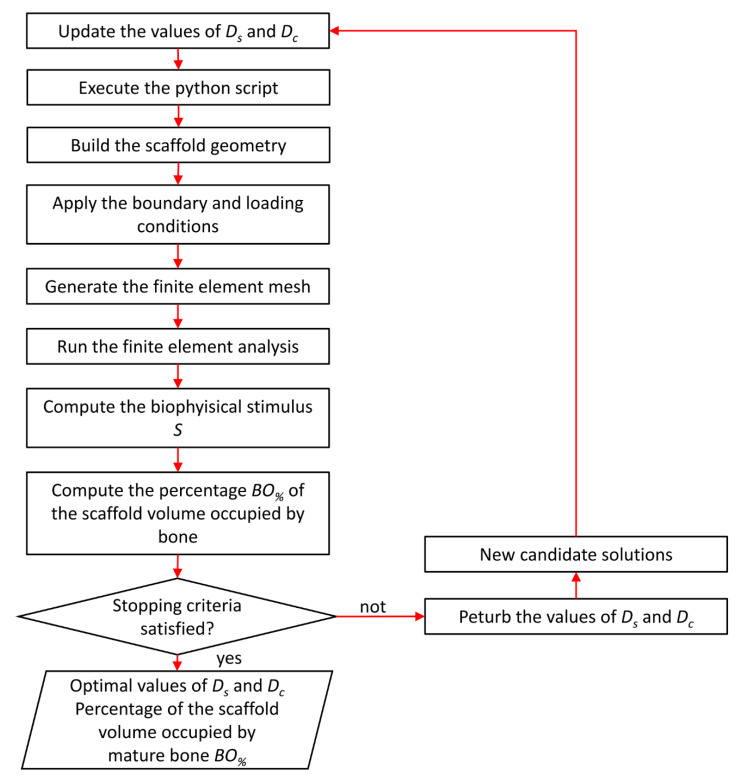
Schematic of the optimization algorithm implemented to determine the optimal scaffold geometry.

The optimization algorithm aims to identify the scaffold geometry that allows maximizing the amounts of neo-formed bone for each value of force per unit area *F_UA_* hypothesized in the study (Figure 6).

In detail, the algorithm, written in Matlab, employs the *fmincon* function from the Matlab optimization toolbox to determine the optimal values of the design variables *D_s_* and *D_c_* that maximize *BO_%_*, the percentage of the scaffold volume occupied by mature bone. In each optimization cycle, the values of *D_s_* and *D_c_* are perturbed and entered into a python script. This script is given in input to Abaqus, which builds the model, applies the boundary and loading conditions, generates the mesh, and runs the finite element analysis. Then, the algorithm reads the results of the FEM analysis, computes the biophysical stimulus *S,* and compares it with the boundary values reported in the inequalities (5). At this point, it computes *BO_%_*, the percentage of the scaffold volume occupied by mature bone, as the ratio between the volume of the elements with *S* that satisfy the inequality 0.01 < *S* < 0.53, and the total volume of the scaffold *L* × *L* × *L*. The algorithm perturbs so many times the values of *D_s_* and *D_c_* until the maximum value of *BO_%_* is determined. Once this occurs, the optimization algorithm stops and outputs the predicted optimal values of the design variables *D_s_* and *D_c_* as well as the value of the percentage *BO_%_*, which represents the maximum percentage of the scaffold volume that can be occupied by bone for a given load value. During the optimization process, *D_s_* and *D_c_* can assume variable values concerning both (L) and (S) Topology but must always satisfy the constraint equations summarized in Table 1.

All the optimization analyses were conducted on an HP XW6600-Intel^®^Xeon^®^DualProcessor E5-5450 3 GHz–32 Gb RAM workstation (Intel Corporation, Mountain View, CA, USA) and required approximately 1500 h of computation.

## 3. Results and Discussion

**Figure 7:**
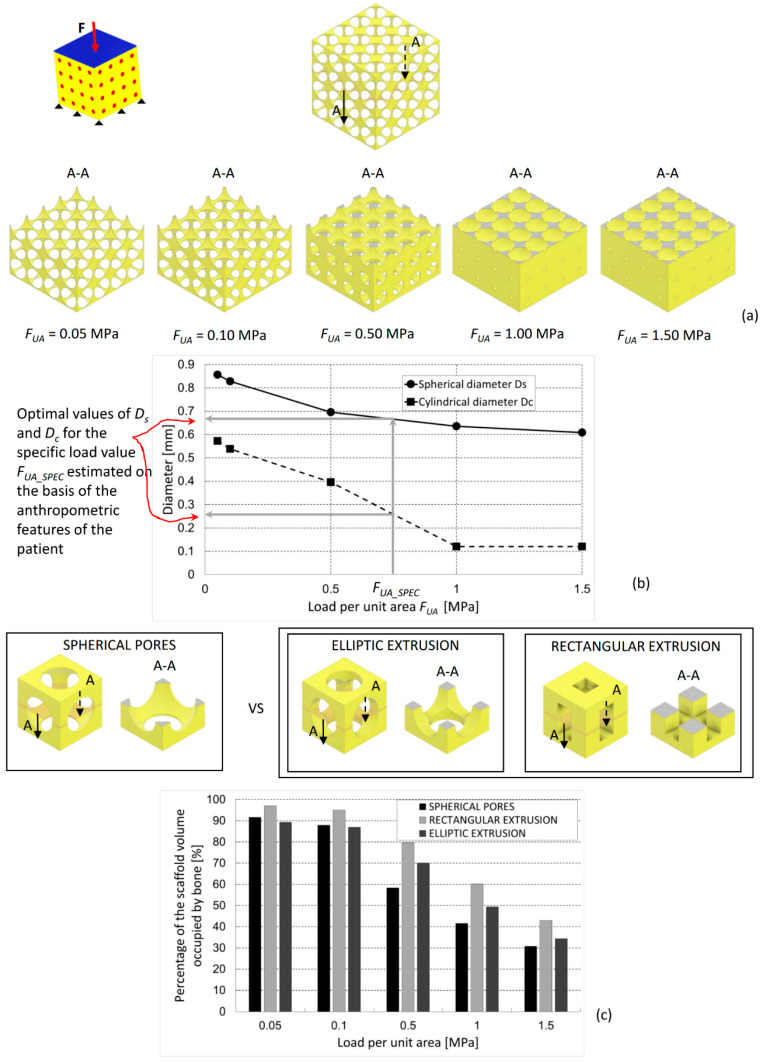
(**a**) Optimized scaffold geometries (section views A-A), (**b**) optimal values of *D_s_* and *D_c_*, and (**c**) percentage of the scaffold volume occupied by mature bone, predicted by the optimization algorithm for different values of the compression load. The percentages of bone are compared with those predicted for scaffolds with hexahedron unit cells including elliptic and rectangular extrusions [20].

The optimized scaffold geometries predicted by the proposed algorithm in the case of compression load present spherical pores and cylindrical interconnections that become smaller for increasing values of the load (Figure 7). This can be explained with the argument that as the load increases, the biophysical stimulus acting on the mesenchymal tissue increases too, thus favoring the formation of soft tissues like cartilage and fibrous tissue. Hence, the algorithm to counterbalance this tends to increase the scaffold stiffness by decreasing the dimensions of the spherical pores and the cylindrical connections (Figure 7a,b). Comparing the percentages *BO_%_* with those predicted in a previous study [20] for regular scaffolds based on a hexahedron unit cell with elliptic and rectangular extrusions, we found that scaffolds with rectangular extrusions perform always better than those with spherical pores. Conversely, those with elliptic extrusions work better than the scaffolds with spherical pores only for high load values (Figure 7c). When the load is high, in fact, elliptic and rectangular extrusions tend to orientate according to the load direction, which makes the scaffold more “suited” to bear and transfer the compression load acting on it.

**Figure 8:**
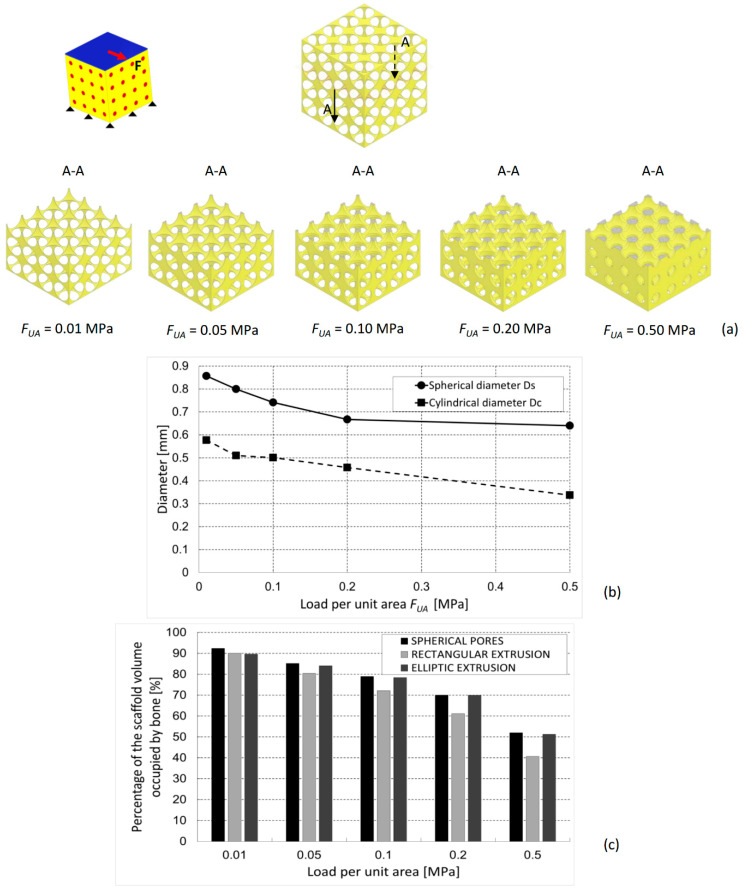
(**a**) Optimized scaffold geometries (section views A-A), (**b**) optimal values of *D_s_* and *D_c_*, and (**c**) percentage of the scaffold volume occupied by mature bone, predicted by the optimization algorithm for different values of the shear load. The percentages of bone are compared with those predicted for scaffolds with hexahedron unit cells including elliptic and rectangular extrusions [20].

The optimal geometries predicted in the case of shear load present pores with dimensions that get increasingly smaller as we move towards higher load values (Figure 8a,b). Interestingly, in this case, the scaffold with spherical pores performs, for all the hypothesized values of shear load, better than those with elliptic and rectangular extrusions (Figure 8c).

**Figure 9:**
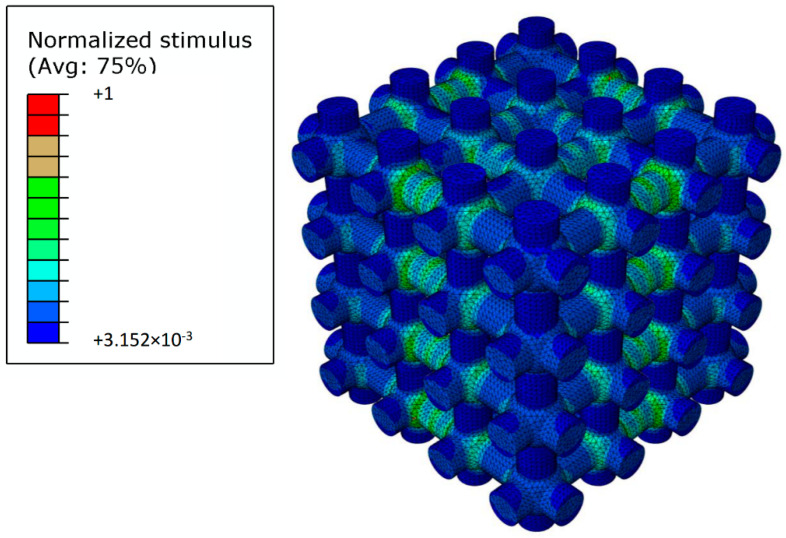
Spatial distribution of the normalized biophysical stimulus *S*/*S_max_* computed for a scaffold (*D_s_* = 0.425 mm *D_c_* = 0.275 mm) subjected to the compression load of *F_UA_* = 0.5 MPa.

In general, the biophysical stimulus *S* acting on the mesenchymal tissue assumes higher values in the proximity of the spherical pores, while smaller values are observed in the proximity of the cylindrical interconnections (Figure 9). The regularity of the scaffold geometry leads to a regular distribution of the biophysical stimulus that is repeated with approximately the same characteristics as many times as the cells of the scaffold. Such a spatial distribution demonstrates that the biophysical stimulus depends on the scaffold geometry and on how this transfers the load to the mesenchymal tissue.

The proposed study has some limitations. First, the model includes a spherical pore the diameter of which was optimized based on the mechanobiological model of Prendergast et al. [14]. As demonstrated in previous studies [16,20], scaffolds oriented according to the load direction perform better than those without a specific orientation [18]. To make the proposed geometry “oriented” according to the load direction, the spherical surface should be changed with that of prolate or oblate spheroids. In this case, the number of variables to optimize are two: the minor and the major axis of the spheroid. With this strategy, the spheroidal surface would properly orient, thus making the scaffold more “suited” to bear and transfer the load acting on it [22,23,24]. This topic will be the objective of future studies. Second, a clear and direct experimental study that demonstrates the correctness of the predictions of the proposed model is, at the moment, lacking. In general, it is difficult to systemically study the effects of scaffold geometry on the process of bone tissue regeneration. The identification of the geometrical features that principally affect the tissue differentiation process occurring in a scaffold requires the systematic study of different scaffold geometries. However, at the moment, no such studies are available in the literature [7]. Third, a simplified hypothesis was followed regarding the diffusion of mesenchymal stem cells once the scaffold is implanted. The event in which the MSCs migrate from the adjacent tissues and invade the scaffold could not take place *sic et simpliciter*. In fact, once a scaffold is implanted, it will be most likely infiltrated with blood, which clots within a few minutes, thus clogging the pores of the scaffolds. Moreover, other cells such as connective tissue fibroblasts could compete with MSCs to colonize the scaffolds. However, in the case where MSCs are the only cells entering the scaffold, having a highly osteogenic microarchitecture, once the new bone is deposited, it will prevent further MSCs inwards migration and bone ingrowth. Studies on the transient phase of the MSCs migration and diffusion through the scaffold should be carried out in the future. Fourth, the proposed algorithm allows to determine the optimal dimensions of the spherical pores and the cylindrical interconnections. However, this poses relevant technological issues in the sense that the proposed approach requires the implementation of additive manufacturing techniques that must guarantee adequate precision for the produced scaffolds. Stereolithography is one of the most powerful and versatile additive manufacturing techniques [25]. It has the highest fabrication accuracy, which ranges from 1.2 to 200 µm [26]. Fused deposition modelling (FDM) was demonstrated to have the lowest precision [27]. The experimental tests previously conducted with FDM demonstrated that this technique is suitable to build accurate scaffold samples only in the cases where the strand diameter is close to the nozzle diameter. Conversely, when a large difference exists, large fabrication errors can be committed on the diameter of the filaments [17]. Scaffolds fabricated with selective laser sintering (SLS) show dimensional deviations—with respect to the nominal dimensions—up to 7.5% [28]. Fifth, the scaffold model investigated has rather small dimensions with respect to those of the scaffolds commonly used in the clinical context. In principle, using a larger scaffold model is possible but poses serious issues of computational power. Sixth, the time variable was not included in the proposed algorithm, i.e., we do not simulate how the bone regeneration process takes place in the scaffold and optimize the scaffold geometry based on the “picture” taken at the instant of time zero, after its implantation. In reality, the inclusion of the time variable requires very high computational power and a computational time tremendously longer than the time required to perform the optimization analyses carried out in this study. In fact, for each candidate geometrical solution, the algorithm should ideally predict how the bony tissue growths and how the scaffold dissolves. This series of analysis cycles should be repeated as many times as the cycles required by the optimization algorithm, which leads to computational times at least two orders of magnitude larger than those required in this study. Increases in computational power will ultimately allow simulating the bone regeneration and the scaffold dissolution processes to optimize the scaffold geometry on a temporal perspective as well as modelling scaffolds with dimensions closer to those actually employed in clinical practice.

**Figure 10:**
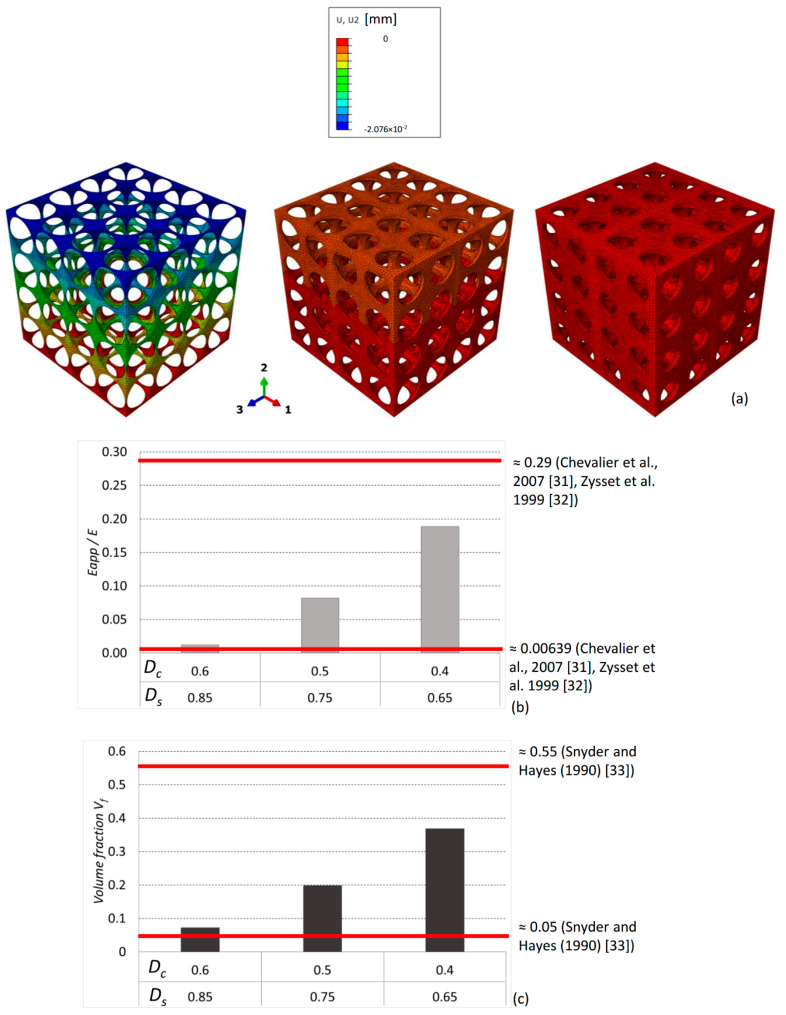
(**a**) *u*_2_ displacement field of the scaffold models subjected to a compression load of 0.1 MPa. (**b**) Values of the ratio *E_app_* /*E* computed for the three models and compared with those experimentally measured (represented with the red lines) for cancellous bone. (**c**) Scaffold volume fraction values compared with the volume fraction of human spongy bone.

Despite these limitations, the proposed model shows a mechanical behavior consistent with that of spongy bone. In fact, if we compute the ratio *E_app_* /*E*, where *E_app_* is the “apparent” Young’s modulus of the scaffold considered in its entirety and *E* = 1000 MPa is Young’s modulus of the material the scaffold is made from (Table 2), we find values falling within the variability range of this ratio experimentally measured for cancellous bone (Figure 10).

To compute the ratio *E_app_* /*E*, three different finite element models of the sole scaffold (i.e., the granulation tissue was removed) were built, with the following pairs of *D_s_* and *D_c_* values expressed in millimeters [mm]: (*D_s_* = 0.85; *D_c_* = 0.55), (*D_s_* = 0.75; *D_c_* = 0.5), (*D_s_* = 0.65; *D_c_* = 0.45), which are close to the typical dimensions of pores commonly adopted in scaffolds for bony tissue [29,30]. These models were clamped on the lower base and subjected to a compression load of *F_UA_* = 0.1 MPa. The displacement *u*_2_ (Figure 10a) produced by the load was computed with Abaqus and used to determine the apparent Young’s modulus as:

*E_app_* = *F_UA_* × *L*/*u*_2_, (6)

Interestingly, the values of the ratio predicted numerically are consistent with those measured experimentally [31,32] on samples of human spongy bone (Figure 10b). Furthermore, if we compute for the three models described above the scaffold volume fraction *V_f_*, i.e., the ratio between the volume of the scaffold *V_s_* and the total volume of the model *V_tot_* = *L × L × L*, we find values that are consistent with those experimental reported by Snyder and Hayes [33] and measured for human spongy bone (Figure 10c).

The proposed model fits well the requirements of so-called Precision Medicine. The optimization algorithm presented in this article represents a possible approach to try to identify, given the specific patient with her/his specific anthropometric characteristics (i.e., macroscopic characteristics of the patient, such as weight, height, and geometric parameters of posture, that is, all the characteristics that allow identifying the boundary and loading conditions that act on a given anatomical region when a specific activity is performed), which are the optimal dimensions of the scaffold micro-geometry to achieve a successful follow-up with the formation of the largest amounts of bone in the shortest possible time? In fact, if one knows the anthropometric characteristics of the patient, they can hypothesize the possible value of load acting on the scaffold that will be implanted, and through diagrams such as those shown in Figure 7b and Figure 8b, they can determine the optimal dimensions of the scaffold that favor the formation of the largest amounts of bone (Figure 7b). Furthermore, the proposed approach can support the surgeon in the choice of the best scaffold to implant in the specific fracture site of the patient. In fact, the surgeon has nowadays a very large range of scaffold geometries available on the market and hence has to choose the most suitable one for the specific requirements of the patient. For example, if, based on the anthropometric characteristics and the anatomical region of the fracture site, it is found that the scaffold will be subjected mainly to compression loading, the surgeon will choose the scaffold with rectangular extrusions (Figure 7c). If, on the other hand, it is found that the acting load will be mainly shear, then the surgeon will choose the scaffold with spherical pores (Figure 8c).

## 4. Conclusions

In this study, using a mechanobiology-based optimization algorithm, we computed the optimal dimensions of the micro-architecture of scaffolds including spherical pores and cylindrical interconnections. The optimization algorithm perturbs the scaffold geometry until the specific dimensions that favor the formation of the largest amounts of bone are identified. The proposed algorithm can guide and support the surgeon in the choice of a “personalized” scaffold that better suits the anthropometric characteristics of the patient, thus allowing to achieve a successful follow-up in the shortest possible time.

## Figures and Tables

**Table 1:** Constraint equations that the diameter of the sphere *D_s_* and the cylinders *D_c_* must satisfy to guarantee the coherence of the scaffold geometry.

Constraint Equation for *D_s_*	Topology	Constraint Equation for *D_c_*
if 0 < *D_s_* ≤ *L_uc_* →	Small topology (S) →	0 < *D_c_* ≤ *D_s_*/$\sqrt{2}$
if *L_uc_* < *D_s_* ≤ *L_uc_* × $\sqrt{2}$ →	Large topology (L) →	$\sqrt{\left(D_{s}^{2}-L_{uc}^{2}\right)}$ < *D_c_* ≤ *D_s_*/$\sqrt{2}$

**Table 2:** Material properties utilized in the model of scaffold and granulation tissue [15,17,18].

Material Property	Granulation Tissue	Scaffold
Young’s modulus [MPa]	0.2	1000
Poisson’s ratio	0.167	0.3
Permeability [m4/(Ns)]	1 × 10^−14^	1 × 10^−14^
Porosity	0.8	0.5
Bulk modulus grain [MPa]	2300	13,920
Bulk modulus fluid [MPa]	2300	2300
